# The IMAGE Statement: A Proposed Reporting Guideline for Clinical Images—A CARE Guideline Extension

**DOI:** 10.3390/mps9040121

**Published:** 2026-08-18

**Authors:** Howard Lopes Ribeiro Junior, Humberto Morais, Fidel Manuel Cáceres-Loriga, Mauer Alexandre da Ascensão Gonçalves

**Affiliations:** 1 Faculty of Medicine, Federal University of Ceará, Fortaleza 60430-160, Ceará, Brazil; 2 Cardiology Department, Hospital Militar Principal Instituto Superior, Luanda, Angola; hmorais1@gmail.com; 3 Doctors HealthCare, Coral Gables, FL 33134, USA; dr.caceres10@hotmail.com; 4 Center for Advanced Studies in Medical Education and Training, Faculty of Medicine, Agostinho Neto University, Luanda 123, Angola; mauergoncalves@gmail.com

**Keywords:** IMAGE Statement, clinical images, reporting guideline, EQUATOR Network

## Abstract

Clinical image publications represent an important educational resource across healthcare disciplines, providing concise visual demonstrations of diseases, diagnostic findings, and therapeutic outcomes. Despite their widespread use, reporting practices remain highly heterogeneous, with substantial variability in the description of clinical context, image acquisition, ethical considerations, and educational content. Existing reporting guidelines, including the CARE Guidelines, do not specifically address the unique methodological and technical aspects of image-based publications. The aim of this study was to develop the IMAGE Statement (Improving Clinical Image Reporting Standards), a reporting guideline specifically designed for clinical image publications as an extension of the CARE Guidelines. The development process was informed by recommendations from the EQUATOR Network and included a literature review, analysis of journal instructions for authors, generation of candidate reporting items, and expert consensus. The resulting IMAGE Statement comprises 15 essential reporting items covering the title, patient information, clinical context, diagnostic assessment, image acquisition, image quality, image selection, image annotation, image description, diagnostic interpretation, final diagnosis, educational message, ethics and consent, artificial intelligence use disclosure, and figure legend. The IMAGE Statement provides a structured framework intended to improve completeness, transparency, reproducibility, educational value, and ethical reporting of clinical image publications across healthcare disciplines.

## 1. Introduction

Clinical images represent a distinctive and highly influential form of scientific communication within the health sciences. Unlike conventional research articles and case reports, clinical image publications rely primarily on visual evidence to illustrate diagnostic findings, disease manifestations, procedural outcomes, anatomical variations, pathological features, and therapeutic responses [1]. Their educational value lies in the ability to communicate complex clinical information rapidly and effectively, facilitating pattern recognition, clinical reasoning, and diagnostic decision-making among healthcare professionals. The widespread adoption of digital imaging technologies, advances in diagnostic modalities, and the expansion of online publishing platforms have further increased the volume, accessibility, and educational impact of clinical image publications across medical specialties [1,2].

Despite their widespread use and educational significance, clinical image publications remain characterized by considerable heterogeneity in reporting practices [3]. Variability in the amount of clinical information provided image descriptions, technical details, figure legends, and ethical reporting standards may limit interpretation, reproducibility, and educational value. Inadequate reporting of image acquisition methods, insufficient clinical context, and inconsistent approaches to informed consent and patient privacy create challenges for authors, reviewers, editors, and readers [4,5].

Over the past two decades, reporting guidelines have become essential tools for improving the quality, transparency, and reproducibility of health research. Initiatives such as STROBE [6], CONSORT [7], and PRISMA [8] have substantially improved reporting standards across diverse study designs. Within the field of clinical imaging, the Clinical and Laboratory Images in Publications (CLIP) principles were proposed in 2012 as a framework to improve the quality, transparency, and reproducibility of published images through recommendations addressing image acquisition, selection, modification, presentation, and interpretation [2]. More recently, the Checklist for Artificial Intelligence in Medical Imaging (CLAIM) was developed to improve the reporting quality of studies involving artificial intelligence in medical imaging. Although CLAIM provides detailed recommendations for AI-based imaging research, its scope is restricted to studies evaluating artificial intelligence systems and does not address the reporting of routine clinical image publications intended primarily for education and scientific communication. Over the subsequent decade, the Case Report (CARE) Guidelines [9] emerged as the leading international reporting standard for case-based medical publications, substantially improving the quality and consistency of case report reporting. In contrast, reporting guidance for clinical image publications has remained largely unchanged since the introduction of the CLIP principles [2].

Given the expanding role of clinical images in medical education and scientific communication, there is a clear need for an updated reporting guideline developed using contemporary consensus-based methodology. Nevertheless, clinical image publications differ substantially from traditional case reports in their objectives, structure, length, and reliance on visual evidence as the primary educational and scientific component (Table 1). As a result, important methodological, technical, educational, and ethical aspects of clinical image reporting remain insufficiently addressed, highlighting the need for dedicated and contemporary reporting guidelines.

The rapid integration of artificial intelligence (AI) into healthcare has also transformed the way clinical images are analyzed, interpreted, and utilized in medical practice and research [10]. AI-assisted tools are increasingly being applied to support image interpretation, pattern recognition, diagnostic classification, prognostic assessment, and clinical decision-making across multiple specialties [11]. As these technologies become more prevalent, ensuring transparency regarding the role of AI in image analysis, interpretation, annotation, and reporting has become increasingly important [12]. The growing intersection between clinical imaging and AI further underscores the need for contemporary reporting standards capable of supporting transparency, reproducibility, and scientific integrity in image-based publications [13].

The aim of this initiative was to develop the IMAGE Statement (Improving Clinical Image Reporting Standards), a reporting guideline designed specifically for clinical image publications. Developed as an extension of the CARE Guidelines, the IMAGE Statement seeks to establish evidence-informed recommendations that enhance the completeness, transparency, educational value, reproducibility, and ethical reporting of clinical images across healthcare disciplines. By providing a standardized framework for authors, reviewers, editors, and publishers, the IMAGE Statement aims to improve the quality and consistency of clinical image publications while preserving the concise and visually focused nature that characterizes this important form of scientific communication.

## 2. Methods

### 2.1. Study Design

The development of the IMAGE Statement (Improving Clinical Image Reporting Standards) followed internationally recognized methodological principles for the creation of health research reporting guidelines. The study design was informed by recommendations from the EQUATOR Network and by established approaches used in the development of reporting guidelines such as CARE [9], CONSORT [7], STROBE [6], and PRISMA [8]. The development process comprised three sequential phases: (1) a review of journal instructions for authors, (2) generation and iterative refinement of candidate reporting items based on the literature review and journal analysis, and (3) development of the final IMAGE checklist. The objective was to establish an evidence-informed reporting guideline specifically designed for clinical image publications, addressing methodological, educational, technical, and ethical aspects of image-based scientific communication.

### 2.2. Literature Review of Journal Instructions for Authors

Journals were identified through a structured process involving searches of publisher and journal websites, complemented by bibliographic and indexing resources and manual searches of journal websites. The objective was to identify journals publishing dedicated image-based article formats rather than to perform an exhaustive systematic inventory of all journals publishing clinical images. Eligible journals were those maintaining a dedicated publication category or section for clinical images or equivalent visual formats, including categories such as “Clinical Images”, “Images in Medicine”, “Images in Clinical Medicine”, “Images in Dermatology”, “Clinical Picture”, “Medical Images”, “Image Challenge”, “Diagnostic Images”, “Visual Vignettes”, “Image Gallery”, or equivalent formats.

Following the initial identification, journal eligibility was assessed according to the predefined criterion of having a dedicated image-based publication format. For each eligible journal, information was extracted from the publicly available author instructions regarding manuscript structure, word limits, figure requirements, image specifications, patient consent policies, ethical requirements, authorship criteria, peer-review procedures, and educational objectives. The final sample comprised 54 eligible journals across multiple medical specialties. The journal sample was intentionally expanded to improve the representation of different clinical disciplines and image-based publication formats. The complete list of included journals and their characteristics is provided in Supplementary Table S1.

The purpose of this process was to identify recurring editorial requirements that could inform development of the IMAGE checklist rather than generate an exhaustive inventory of all journals publishing clinical image reports.

### 2.3. Development of the IMAGE Checklist

The final IMAGE checklist was developed through the integration of evidence obtained from the literature review, findings from the structured review of journal instructions for authors, and iterative refinement of candidate reporting items by the authors. Candidate items were organized into thematic domains reflecting the essential components of high-quality clinical image reporting and were refined to improve clarity, applicability, and consistency while preserving the concise nature of clinical image publications. Emphasis was placed on preserving the concise nature of clinical image publications while ensuring sufficient reporting completeness to maximize educational utility, transparency, and reproducibility.

The resulting IMAGE Statement comprises a consensus-based set of reporting recommendations intended for use by authors, peer reviewers, editors, publishers, and academic institutions involved in the preparation, evaluation, and dissemination of clinical image publications. The checklist was designed to be applicable across multiple healthcare disciplines and adaptable to diverse image-based publication formats.

The IMAGE Statement was intentionally designed as a universal reporting framework applicable across multiple clinical imaging modalities, including clinical photography, radiology, pathology, endoscopy, ultrasound, microscopy, and other image-based disciplines. Rather than prescribing modality-specific technical standards, the checklist encourages authors to report the acquisition parameters, annotation methods, and technical information that are most relevant to the specific imaging modality under study.

## 3. Results

### 3.1. Literature Review of Journals Instructions

A review of the author’s instructions from 54 journals publishing image-based articles was conducted to identify common editorial requirements and reporting expectations (Table S1). The journals included dedicated publication formats such as Clinical Images, Images in Medicine, Images in Clinical Medicine, Clinical Picture, Medical Images, Image Challenge, and other short visual report categories spanning multiple medical specialties. Considerable heterogeneity was observed regarding manuscript structure, word limits, permitted numbers of figures, authorship limits, abstract requirements, image annotation standards, image quality specifications, and patient consent policies.

Most journals required concise manuscripts, with word limits most commonly ranging from 250 to 500 words, and 46 out of the 54 journals did not require an abstract. Likewise, most journals limited submissions to one or two figures, although some accepted multiple images or supplementary videos. Access models also varied substantially, with hybrid publication being the most frequent model, followed by fully open-access journals. Despite these editorial differences, several core reporting domains were consistently represented across journal instructions, including patient information, clinical context, image description, diagnostic interpretation, educational value, and ethical considerations. These findings provided practical editorial insights that complemented the literature review and contributed to the construction of the proposed IMAGE checklist. Based on these findings, a preliminary version of the IMAGE checklist was developed, as described in the following section.

### 3.2. Development of the Preliminary IMAGE Checklist

The preliminary IMAGE checklist (Section 4) was developed through an iterative process informed by the literature review, journal analysis, and the collective editorial and academic experience of the authors. Potential reporting domains were identified from recurring themes observed across the literature and journal requirements. Candidate items were subsequently refined to improve clarity, applicability, and relevance to clinical image publications while preserving the concise nature of this publication format.

Particular emphasis was placed on domains that were consistently underreported or inconsistently addressed in published clinical image reports, including image acquisition, image quality, image description, educational messaging, figure legends, and ethical considerations. The resulting checklist represents a proposed framework intended to improve reporting completeness, transparency, educational value, and ethical integrity in clinical image publications.

## 4. The IMAGE Checklist Version 1.0

The IMAGE Statement (Improving Clinical Image Reporting Standards) comprises 12 core reporting items considered essential for the transparent, educational, ethical, and reproducible publication of clinical images. These items were identified through evidence synthesis, review of journal requirements, and expert consensus. The checklist is intended to guide authors in preparing high-quality clinical image reports while supporting editors, peer reviewers, and publishers in evaluating manuscript completeness and reporting quality. The checklist was designed to be applicable across medical, dental, nursing, allied health, and biomedical disciplines. The IMAGE Checklist Version 1.0 is presented in Table 1.

### 4.1. Item 1: Title

The title should clearly identify the manuscript as a clinical image report and accurately reflect the principal finding, diagnosis, or educational message illustrated by the image. Titles should be concise, informative, and facilitate indexing and retrieval.

### 4.2. Item 2: Patient Information

Authors should provide relevant demographic and clinical characteristics necessary for understanding the image findings. Information may include age, sex, relevant medical history, risk factors, and other contextual details directly related to the clinical presentation. For clinical photographs in which appearance may be influenced by skin pigmentation, authors should consider reporting skin phototype (e.g., Fitzpatrick skin type) or other relevant pigmentation characteristics when clinically relevant. Only information essential to interpretation should be reported, and patient confidentiality must be preserved.

### 4.3. Item 3: Clinical Context

A concise description of the clinical scenario should be provided, including presenting symptoms, clinical signs, indication for imaging or examination, and circumstances leading to image acquisition. The objective is to establish sufficient context to support interpretation without converting the report into a full case report.

### 4.4. Item 4: Diagnostic Assessment

Authors should summarize the diagnostic evaluation relevant to the image, including physical examination findings, laboratory investigations, imaging studies, pathology results, or other diagnostic procedures that contributed to clinical decision-making. Information should be limited to findings necessary to understand the significance of the image.

### 4.5. Item 5: Image Acquisition

The method used to obtain the image should be described when relevant to interpretation. This may include imaging modality, acquisition techniques, equipment specifications, magnification, staining procedures, imaging protocols, software used for image processing, and where applicable, relevant acquisition metadata (e.g., device specifications, imaging parameters, DICOM-derived technical information, or acquisition date when scientifically relevant). Such information should be reported when it contributes to transparency, reproducibility, or interpretation, while respecting patient privacy and journal policies.

### 4.6. Item 6: Image Quality

Images should be presented at sufficient resolution and quality to allow for accurate visualization of clinically relevant findings. Authors should ensure that submitted images comply with the technical requirements of the target journal or imaging modality. When scientifically relevant, technical image characteristics (e.g., pixel dimensions, resolution, color space, compression format, bit depth, or other acquisition parameters) should be reported. The IMAGE Statement intentionally does not prescribe universal technical thresholds (such as minimum DPI), because these requirements vary substantially across imaging modalities, acquisition systems, and journal policies. Authors should avoid inappropriate image manipulation and ensure that any adjustments to brightness, contrast, color balance, or other image parameters are applied uniformly and do not alter the scientific content of the image.

### 4.7. Item 7: Image Selection

Authors should indicate whether the image represents a typical, atypical, rare, or particularly illustrative presentation of the condition. When appropriate, the rationale for selecting the image should be provided, emphasizing its clinical relevance, educational value, or contribution to disease recognition and understanding. Reporting the reasons for image selection helps readers assess the representativeness and significance of the visual findings.

### 4.8. Item 8: Image Annotation

Annotations should be used when necessary to facilitate the identification and interpretation of relevant findings. These may include arrows, labels, outlines, symbols, inset panels, scale bars, or other visual indicators that guide the reader to structures, abnormalities, or regions of interest. All annotations should be clearly explained in the figure legend and should not obscure or alter the scientific content of the image.

### 4.9. Item 9: Image Description

A detailed and objective description of the visual findings should be provided. Descriptions should focus on observable features rather than diagnostic conclusions and should identify structures, abnormalities, patterns, or other relevant findings visible in the image. When appropriate, arrows, labels, or annotations may be used to facilitate interpretation.

### 4.10. Item 10: Diagnostic Interpretation

Authors should explain the clinical significance of the image findings and their contribution to diagnosis, differential diagnosis, management, or understanding of the disease process. Interpretation should be evidence-based and clearly linked to the visual findings presented.

### 4.11. Item 11: Final Diagnosis

The confirmed diagnosis or most probable diagnosis should be explicitly stated. When diagnostic uncertainty exists, authors should describe the rationale supporting the proposed diagnosis and discuss relevant alternative considerations.

### 4.12. Item 12: Educational Message

Each clinical image report should include a concise educational message summarizing the principal learning point. This statement should highlight clinical relevance, diagnostic value, or practical implication of the image for healthcare professionals and learners.

### 4.13. Item 13: Ethics and Consent

Authors should confirm that appropriate ethical requirements have been fulfilled, including informed consent for publication whenever applicable. Measures taken to protect patient privacy, confidentiality, and anonymity should be reported. When clinical photographs contain potentially identifiable features, such as the face, tattoos, distinctive scars, birthmarks, jewelry, or other unique characteristics, authors should describe the anonymization measures adopted whenever appropriate, ensuring that such modifications do not compromise the scientific or educational value of the image. Ethical approval requirements should be disclosed according to local regulations and journal policies.

### 4.14. Item 14: AI Use Disclosure

Authors should disclose whether artificial intelligence (AI) tools were used during image acquisition, analysis, interpretation, annotation, enhancement, or manuscript preparation. When AI-assisted methods are employed, the specific tool, software, or model should be identified, and its role clearly described. Transparent reporting of AI use promotes reproducibility, accountability, scientific integrity, and appropriate interpretation of the reported findings.

### 4.15. Item 15: Figure Legend

Each image should be accompanied by a complete, accurate, and self-contained figure legend. The legend should describe the image content, identify relevant structures or findings, explain annotations, and provide sufficient information for readers to interpret the image independently of the main text.

### 4.16. IMAGE Checklist Summary

**Figure 1:**
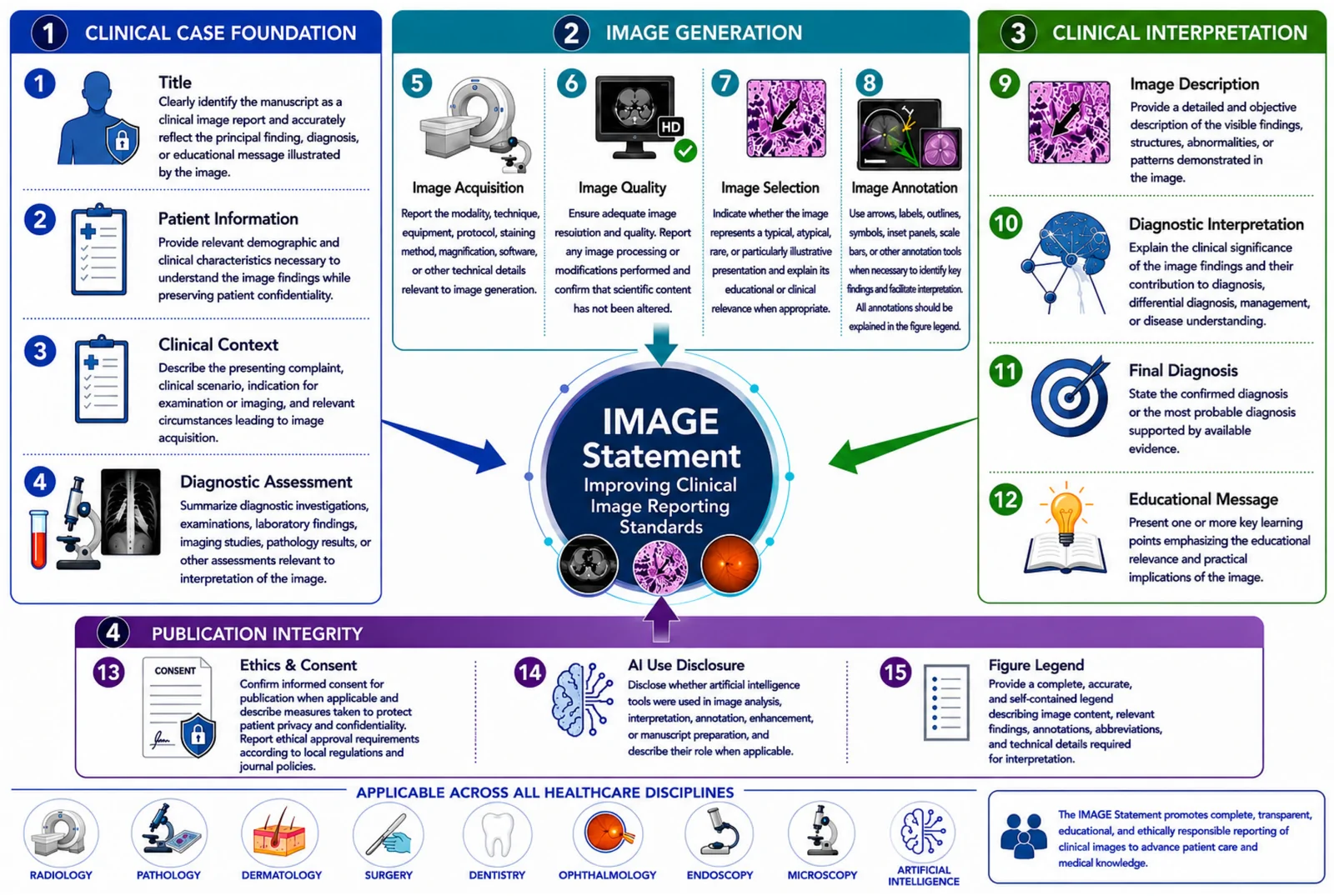
Conceptual framework and reporting domains of the IMAGE Statement (Improving Clinical Image Reporting Standards) Version 1.0. Legend: The IMAGE Statement provides a structured reporting framework for clinical image publications across healthcare disciplines, organized into four major domains comprising 15 essential reporting items. Domain 1 (Clinical Case Foundation) includes Title, Patient Information, Clinical Context, and Diagnostic Assessment, establishing the clinical background necessary for interpretation of image findings. Domain 2 (Image Generation) encompasses Image Acquisition, Image Quality, Image Selection, and Image Annotation, promoting transparency, technical rigor, reproducibility, and optimal visualization of clinically relevant findings. Domain 3 (Clinical Interpretation) includes Image Description, Diagnostic Interpretation, Final Diagnosis, and Educational Message, ensuring an accurate description of visual findings, appropriate clinical interpretation, diagnostic clarity, and educational value. Domain 4 (Publication Integrity) comprises Ethics and Consent, AI Use Disclosure, and Figure Legend, reinforcing ethical responsibility, transparency regarding artificial intelligence utilization, and comprehensive image documentation. Collectively, these domains aim to improve the completeness, consistency, transparency, reproducibility, educational impact, and ethical reporting of clinical images in medicine, dentistry, nursing, allied health, pathology, radiology, surgery, ophthalmology, endoscopy, microscopy, dermatology, and other biomedical disciplines. This conceptual illustration was developed with the assistance of ChatGPT (OpenAI, GPT-5.5) and BioRender and was subsequently reviewed, refined, and approved by the authors.

The IMAGE Statement recommends that all clinical image publications report information addressing the following domains: title, patient information, clinical context, diagnostic assessment, image acquisition, image quality, image selection, image annotation, image description, diagnostic interpretation, final diagnosis, educational message, ethics and consent, AI use disclosure, and figure legend (Table 1 and Figure 1). Collectively, these items aim to improve the completeness, transparency, reproducibility, educational value, and ethical reporting of clinical image publications across healthcare disciplines.

## 5. Explanation of Checklist Items

The IMAGE Statement (Table 1 and Figure 1) is intended to improve the completeness, transparency, educational value, and ethical reporting of clinical image publications. The following section provides the rationale, recommendation, and an illustrative example for each checklist item. This explanatory guidance is intended to assist authors, reviewers, and editors in the consistent application of the IMAGE recommendations across healthcare disciplines.

### 5.1. Item 1: Title

#### 5.1.1. Rationale

The title serves as the first point of communication between authors and readers and plays a critical role in indexing, discoverability, and information retrieval. A clear title allows readers to immediately recognize the manuscript as a clinical image report and identify its principal educational or diagnostic focus.

#### 5.1.2. Recommendation

Titles should explicitly identify the report as a clinical image, image challenge, clinical picture, or equivalent format according to journal requirements. The title should accurately reflect the principal finding, diagnosis, or educational message without overstating conclusions.

#### 5.1.3. Example

Clinical Image: Oral Melanoma Presenting as an Asymptomatic Pigmented Palatal Lesion.

### 5.2. Item 2: Patient Information

#### 5.2.1. Rationale

Clinical interpretation of visual findings requires an understanding of relevant patient characteristics. However, excessive detail may compromise confidentiality without contributing to educational value.

#### 5.2.2. Recommendation

Provide only demographic and clinical information necessary to understand the image findings. Relevant details may include age, sex, medical history, risk factors, or clinical background directly related to the image. For clinical photographs in which skin pigmentation may influence interpretation, authors are encouraged to report skin phototype (e.g., Fitzpatrick skin type) or other relevant pigmentation characteristics whenever available and clinically relevant.

#### 5.2.3. Example

A 67-year-old male with a 40-pack-year smoking history presented with a persistent ulcerative lesion of the lateral tongue.

### 5.3. Item 3: Clinical Context

#### 5.3.1. Rationale

Images interpreted in isolation may be misleading or difficult to understand. Brief clinical context facilitates interpretation and enhances educational relevance.

#### 5.3.2. Recommendation

Describe the presenting symptoms, clinical findings, indication for examination or imaging, and circumstances that led to image acquisition.

#### 5.3.3. Example

The lesion had been progressively enlarging for over six months and was associated with intermittent pain during mastication.

### 5.4. Item 4: Diagnostic Assessment

#### 5.4.1. Rationale

Clinical images rarely represent the entirety of the diagnostic process. Readers benefit from understanding how the image contributed to diagnosis and clinical decision-making.

#### 5.4.2. Recommendation

Summarize relevant diagnostic investigations, including laboratory findings, imaging studies, histopathological examination, or other assessments necessary for interpretation.

#### 5.4.3. Example

Incisional biopsy demonstrated invasive squamous cell carcinoma with keratin pearl formation.

### 5.5. Item 5: Image Acquisition

#### 5.5.1. Rationale

The interpretation and reproducibility of visual findings may depend on the image acquisition methods. Reporting technical details enhances transparency and scientific rigor.

#### 5.5.2. Recommendation

Describe the modality, technique, equipment, magnification, staining method, acquisition protocol, software, and when relevant, acquisition metadata that contribute to image interpretation or reproducibility. Examples include imaging parameters, device specifications, and DICOM-derived technical information. Metadata should be reported only when scientifically relevant and consistent with institutional privacy requirements and journal policies.

#### 5.5.3. Example

The CT examination was acquired using a 128-slice multidetector scanner (120 kVp, automatic tube current modulation, 1-mm slice thickness). Relevant DICOM acquisition parameters were reviewed to confirm image integrity and are reported as applicable. Patient-identifying metadata were removed in accordance with institutional privacy policies.

### 5.6. Item 6: Image Quality

#### 5.6.1. Rationale

Image quality directly influences interpretation, educational effectiveness, and scientific credibility. Poor-quality images may obscure important findings and reduce reproducibility.

#### 5.6.2. Recommendation

Ensure adequate resolution and visual clarity according to the requirements of the imaging modality and the target journal. When technically or scientifically relevant, report image characteristics such as resolution, pixel dimensions, color space, compression format, bit depth, or other acquisition parameters. Universal technical thresholds are intentionally not specified because appropriate image quality standards differ among imaging modalities and publishers. Report any post-processing procedures and avoid manipulations that alter the scientific content.

#### 5.6.3. Example

Brightness and contrast were adjusted uniformly across the image without modification of anatomical structures or pathological findings.

### 5.7. Item 7: Image Selection

#### 5.7.1. Rationale

The educational and scientific value of a clinical image depends not only on its quality, but also on its relevance and representativeness. Readers should understand why a particular image was selected for publication and whether it represents a typical, unusual, rare, or particularly illustrative manifestation of a condition. Transparent reporting of image selection enhances educational value and reduces the risk of selection bias.

#### 5.7.2. Recommendation

Indicate whether the image represents a typical, atypical, rare, or particularly illustrative presentation. When relevant, explain the rationale for selecting the image and its educational or clinical significance.

#### 5.7.3. Example

This image was selected because it demonstrates a characteristic radiographic appearance of odontogenic keratocyst with well-defined corticated borders and minimal buccolingual expansion, representing a typical presentation of the condition.

### 5.8. Item 8: Image Annotation

#### 5.8.1. Rationale

Annotations facilitate the identification of relevant structures and findings, guiding readers to the features intended for educational or diagnostic emphasis and reducing ambiguity in image interpretation.

#### 5.8.2. Recommendation

Use arrows, arrowheads, labels, outlines, symbols, inset panels, or other annotation tools when necessary to identify key findings. All annotations should be clearly explained in the figure legend and applied in a manner that preserves image readability.

#### 5.8.3. Example

Black arrows indicate the margins of the lesion, while the asterisk identifies the adjacent anatomical structure used as a reference.

### 5.9. Item 9: Image Description

#### 5.9.1. Rationale

Objective description enables readers to independently evaluate visual findings before considering diagnostic interpretation.

#### 5.9.2. Recommendation

Describe observable structures, abnormalities, patterns, and features visible in the image. Avoid embedding diagnostic conclusions within the descriptive section.

#### 5.9.3. Example

The lesion appears as an irregularly bordered pigmented area measuring approximately 18 mm in diameter with heterogeneous coloration and focal ulceration.

### 5.10. Item 10: Diagnostic Interpretation

#### 5.10.1. Rationale

Visual findings acquire educational significance when linked to clinical meaning and diagnostic reasoning.

#### 5.10.2. Recommendation

Explain the significance of the image findings and their relationship to diagnosis, differential diagnosis, management, or disease pathophysiology.

#### 5.10.3. Example

The asymmetry, irregular pigmentation, and ulcerative component raised suspicion for malignant melanoma, prompting histopathological confirmation.

### 5.11. Item 11: Final Diagnosis

#### 5.11.1. Rationale

Readers should be informed of the definitive or most probable diagnosis associated with the image.

#### 5.11.2. Recommendation

Clearly state the confirmed diagnosis and the basis for diagnostic confirmation.

#### 5.11.3. Example

Final Diagnosis: Oral mucosal melanoma confirmed by histopathological and immunohistochemical examination.

### 5.12. Item 12: Educational Message

#### 5.12.1. Rationale

The primary purpose of most clinical image publications is educational. Explicit learning points improve knowledge translation and clinical applicability.

#### 5.12.2. Recommendation

Include one or more concise learning points highlighting the relevance of the image to clinical practice.

#### 5.12.3. Example

Learning Point: Persistent pigmented oral lesions with irregular borders should prompt early biopsy to exclude oral melanoma.

### 5.13. Item 13: Ethics and Consent

#### 5.13.1. Rationale

Clinical images may contain identifiable information and therefore require careful consideration of patient rights, privacy, and confidentiality.

#### 5.13.2. Recommendation

State whether informed consent for publication was obtained and describe the measures adopted to preserve patient anonymity. When applicable, report whether facial features or other potentially identifiable characteristics (e.g., tattoos, scars, birthmarks, jewelry, or unique physical features) were masked, cropped, or otherwise anonymized in a manner that preserves the scientific integrity and educational value of the image. Ethical approval requirements should be reported according to institutional and journal policies.

#### 5.13.3. Example

Written informed consent for publication of the clinical information and images was obtained from the patient prior to manuscript submission. Facial features and other potentially identifiable characteristics were appropriately anonymized while preserving the diagnostic content of the image.

### 5.14. Item 14: AI Use Disclosure

#### 5.14.1. Rationale

AI tools are increasingly used to assist image analysis, interpretation, annotation, enhancement, and manuscript preparation. Transparent disclosure of AI involvement is essential to ensure scientific integrity, reproducibility, and appropriate evaluation of the reported findings.

#### 5.14.2. Recommendation

Authors should disclose whether AI tools were used in the analysis, interpretation, annotation, enhancement, or reporting of the clinical image. The name of the tool, its intended purpose, and the extent of its contribution should be described. AI use should not replace human clinical judgment, and authors remain responsible for the accuracy of all reported information.

#### 5.14.3. Example

An artificial intelligence-based software tool was used to assist lesion segmentation and image annotation. All findings were independently reviewed and validated by the treating clinicians before publication.

### 5.15. Item 15: Figure Legend

#### 5.15.1. Rationale

A figure legend should provide sufficient information to allow for interpretation of the image independently of the main text.

#### 5.15.2. Recommendation

Include a complete description of image content, relevant findings, annotations, abbreviations, scale bars, and technical information when appropriate.

#### 5.15.3. Example

Figure 1. Clinical photograph demonstrating an irregularly pigmented lesion involving the hard palate. The lesion exhibits asymmetrical borders, heterogeneous pigmentation, and focal ulceration (arrow), features suggestive of oral melanoma.

### 5.16. Summary

Collectively, the 12 IMAGE checklist items provide a structured framework for reporting clinical image publications. Adherence to these recommendations is expected to improve reporting completeness, educational value, transparency, reproducibility, and ethical compliance while maintaining the concise format characteristic of clinical image reports.

## 6. Discussion

### 6.1. Principal Findings

The IMAGE Statement was developed to address a longstanding gap in health research reporting by providing a dedicated framework for the preparation and publication of clinical image reports. Through a structured process involving evidence synthesis, analysis of journal requirements, and iterative refinement of candidate reporting items, we identified a set of core reporting elements considered essential for the transparent, educational, ethical, and reproducible reporting of clinical images. The IMAGE Statement was intentionally developed as a concise reporting framework that can be adapted to the diverse editorial requirements of journals publishing clinical images. Rather than establishing mandatory and optional reporting categories, the checklist follows the philosophy of EQUATOR reporting guidelines, whereby each item should be addressed whenever applicable to the specific clinical image and publication format. This flexible approach allows authors to balance reporting completeness with journal-specific word limits while preserving the educational and scientific value of clinical image publications.

The final IMAGE checklist comprises 12 reporting items encompassing title, patient information, clinical context, diagnostic assessment, image acquisition, image quality, image description, diagnostic interpretation, final diagnosis, educational message, ethics and consent, and figure legend. Collectively, these domains reflect the unique characteristics of clinical image publications, which differ from traditional case reports by emphasizing visual evidence as the primary vehicle for scientific communication and education.

The development process revealed substantial variability in current reporting practices across journals and specialties. Inconsistencies were particularly evident in the reporting of clinical context, image acquisition methods, image annotations, educational content, and ethical safeguards. The IMAGE Statement was designed to address these deficiencies while preserving the concise format that distinguishes clinical image publications from conventional case reports.

### 6.2. Comparison with the CARE Guidelines and Other Reporting Guidelines

The IMAGE Statement was developed as an extension of the CARE Guidelines [9] and therefore retains several foundational principles that have contributed to the success and widespread adoption of CARE in case report reporting. Both guidelines emphasize transparency, clinical relevance, patient-centered reporting, diagnostic clarity, and ethical responsibility. The inclusion of patient information, clinical context, diagnostic assessment, final diagnosis, and informed consent reflects these shared objectives. However, important modifications were necessary to accommodate the distinctive nature of clinical image publications. Unlike traditional case reports, clinical image reports are generally brief, highly visual, and primarily educational in purpose. Consequently, several components commonly included in CARE reports, such as detailed timelines, therapeutic interventions, longitudinal follow-up, and extensive narrative descriptions, were not incorporated as mandatory elements within the IMAGE framework.

Conversely, the IMAGE Statement introduces reporting domains that are absent from or only indirectly addressed by CARE. These include image acquisition methodology, image quality and integrity, image selection, objective image description, image annotation, educational messaging, disclosure of artificial intelligence use, and comprehensive figure legends. These elements were considered essential because the scientific and educational value of clinical image publications depends fundamentally on the quality, interpretability, and contextualization of visual evidence.

The IMAGE Statement also differs from the Checklist for Artificial Intelligence in Medical Imaging (CLAIM) guideline, which was developed to improve the reporting of studies evaluating artificial intelligence in medical imaging. Whereas CLAIM focuses on AI research methodology and model development, the IMAGE Statement addresses the reporting of clinical image publications across healthcare disciplines, irrespective of imaging modality or artificial intelligence involvement. Accordingly, the two guidelines should be viewed as complementary rather than competing, serving distinct purposes within the scientific reporting ecosystem.

Overall, the IMAGE Statement should not be regarded as a replacement for existing reporting guidelines but rather as a complementary extension specifically designed to address the methodological, technical, educational, and ethical requirements of clinical image publications.

### 6.3. Benefits of the IMAGE Statement

The IMAGE Statement offers potential benefits for multiple stakeholders involved in the creation, evaluation, and dissemination of clinical image publications.

### 6.4. Benefits for Authors

For authors, the guideline provides a structured framework that facilitates manuscript preparation and promotes reporting completeness. By clearly identifying essential reporting elements, IMAGE may reduce omissions, improve educational value, and enhance the likelihood of successful peer review and publication.

### 6.5. Benefits for Reviewers

For peer reviewers, the checklist offers a standardized tool for assessing reporting quality, scientific rigor, image integrity, and ethical compliance. The use of explicit reporting criteria may improve consistency across review processes and facilitate objective manuscript evaluation.

### 6.6. Benefits for Editors and Publishers

For editors and publishers, IMAGE provides a practical framework for establishing editorial standards and harmonizing submission requirements across journals. Adoption of the guideline may contribute to improved reporting quality, greater consistency among published reports, and enhanced trustworthiness of image-based scientific content.

### 6.7. Benefits for Readers and Learners

For readers, educators, and healthcare professionals, improved reporting standards may increase the interpretability, reproducibility, and educational value of clinical image publications. Standardized reporting facilitates knowledge transfer, supports clinical learning, and promotes the accurate interpretation of visual findings across disciplines. Collectively, these benefits may contribute to the advancement of clinical image publications as a reliable and effective form of scientific and educational communication.

### 6.8. Limitations

Several limitations should be considered when interpreting the development and proposed application of the IMAGE Statement. First, although the guideline was informed by a comprehensive review of the literature and journal requirements, the available evidence specifically addressing clinical image reporting remains limited compared with other reporting domains. Consequently, certain recommendations were necessarily informed by expert consensus rather than high-certainty empirical evidence.

Second, the review of journal instructions was conducted as a review intended to identify common reporting requirements rather than as a systematic review designed to capture every journal publishing clinical image reports. Although efforts were made to include journals from multiple healthcare specialties, additional journals meeting the eligibility criteria may exist and could be incorporated in future updates of the IMAGE Statement. Third, despite efforts to achieve multidisciplinary and international representation, the composition of the expert panel may not fully reflect all healthcare disciplines, geographic regions, or publication cultures in which clinical image reports are produced. Future updates may benefit from broader stakeholder engagement and additional specialty-specific input. Fourth, the diversity of image-based publication formats across journals presents an inherent challenge to standardization. Clinical image reports vary considerably with respect to word limits, image requirements, editorial objectives, and peer-review practices. Although the IMAGE Statement was designed to be broadly applicable, some adaptation may be required to accommodate journal-specific requirements.

Finally, the effectiveness of the IMAGE Statement in improving reporting quality remains to be formally evaluated. Future studies should assess guideline uptake, adherence, usability, and impact on publication quality following implementation. In addition, the IMAGE Statement has not yet been formally piloted among practicing clinicians, authors, peer reviewers, or editors. Although the checklist was intentionally designed to be concise and practical, its usability, acceptability, completion time, and perceived usefulness in routine manuscript preparation remain to be evaluated. Future multicenter implementation studies involving end-users from different specialties and healthcare settings will be essential to validate its feasibility and optimize future versions of the guideline.

### 6.9. Future Directions

The IMAGE Statement should be regarded as an evolving reporting framework subject to periodic review and refinement. As clinical imaging technologies, publishing practices, ethical standards, and artificial intelligence continue to evolve, updates to the guideline may be necessary to ensure its ongoing relevance and applicability. Future work should focus on several priorities. First, formal endorsement by journals, publishers, professional societies, and academic institutions may facilitate broader dissemination, implementation, and international adoption of the guideline. Second, the development of a dedicated Explanation and Elaboration (E&E) document may provide additional methodological guidance, practical examples, and implementation recommendations for authors, reviewers, and editors.

Third, future specialty-specific extensions may further refine IMAGE recommendations for individual imaging modalities, including radiology, pathology, dermatology, ophthalmology, endoscopy, ultrasound, microscopy, and other disciplines with distinct technical acquisition and reporting requirements. These extensions may also incorporate specialty-specific recommendations, such as standardized reporting of skin phototype, skin pigmentation characteristics, and diversity considerations in dermatology and other visually oriented specialties, thereby improving educational value, promoting equity, and reducing potential bias in both clinical interpretation and artificial intelligence applications. Fourth, future implementation studies should evaluate the usability, acceptability, adherence, and educational impact of the IMAGE Statement among authors, peer reviewers, editors, and practicing clinicians. These studies should also assess inter-rater agreement, reporting completeness, manuscript quality, peer-review efficiency, and editorial decision-making following guideline implementation.

Finally, future work may explore practical implementation resources, including abbreviated checklists, digital author-support tools, and other strategies to facilitate the application of the IMAGE Statement in journals with restrictive word limits while maintaining reporting completeness and flexibility across different publication formats. Ultimately, widespread international adoption of the IMAGE Statement has the potential to establish a common reporting standard for clinical image publications, thereby enhancing transparency, educational value, scientific rigor, reproducibility, and ethical integrity across the global biomedical literature.

## 7. Conclusions

The IMAGE Statement (Improving Clinical Image Reporting Standards) represents, to our knowledge, the first reporting guideline specifically developed to standardize the preparation, reporting, and publication of clinical image reports across healthcare disciplines. Developed as an extension of the CARE Guidelines through a structured, consensus-based methodology, IMAGE addresses a critical gap in the current reporting guideline landscape by providing recommendations tailored to the unique methodological, educational, technical, and ethical requirements of image-based scientific communication.

By establishing a standardized framework for reporting clinical images, the IMAGE Statement aims to improve the completeness, transparency, reproducibility, educational value, and ethical integrity of clinical image publications. The guideline is intended to serve as a common reporting foundation across imaging modalities while remaining sufficiently flexible to accommodate future specialty-specific extensions. As the volume and influence of image-based publications continue to expand across the biomedical literature, the adoption of standardized reporting practices becomes increasingly important. The IMAGE Statement provides a practical and evidence-informed foundation for harmonizing reporting standards while preserving the concise and visually focused nature that characterizes clinical image publications.

Future validation studies, journal endorsements, and international implementation efforts will be essential to evaluate the impact of IMAGE on reporting quality and to support its continued refinement. Nevertheless, the IMAGE Statement constitutes an important step toward establishing a globally recognized reporting standard for clinical image publications and advancing the quality, consistency, and educational impact of visual evidence in healthcare research and practice.

## Figures and Tables

**Table 1:** The IMAGE Checklist (Improving Clinical Image Reporting Standards).

Section	Item No.	Recommendation	Explanation	Reported on Page
**Title**	1	Clearly identify the manuscript as a clinical image report and accurately reflect the principal finding, diagnosis, or educational message illustrated by the image.	A clear and informative title facilitates indexing, retrieval, and identification of the publication as an image-based report.	
**Patient Information**	2	Provide relevant demographic and clinical characteristics necessary to understand the image findings while preserving patient confidentiality.	Essential patient information enhances interpretation of the image while maintaining privacy and confidentiality.	
**Clinical Context**	3	Describe the presenting complaint, clinical scenario, indication for examination or imaging, and relevant circumstances leading to image acquisition.	Clinical context allows readers to understand the relevance and applicability of the visual findings.	
**Diagnostic Assessment**	4	Summarize diagnostic investigations, examinations, laboratory findings, imaging studies, pathology results, or other assessments relevant to interpretation of the image.	Diagnostic information should support understanding of the image without requiring a complete case report.	
**Image Acquisition**	5	Report the modality, technique, equipment, protocol, staining method, magnification, software, or other technical details relevant to image generation.	Technical details improve transparency, reproducibility, and the interpretation of image findings.	
**Image Quality**	6	Ensure adequate image resolution and quality. Report any image processing or modifications performed and confirm that scientific content has not been altered.	High-quality images are essential for accurate interpretation and educational value. Image manipulation should be transparent and scientifically appropriate.	
**Image Selection**	7	Indicate whether the image represents a typical, atypical, rare, or particularly illustrative presentation and explain its educational or clinical relevance when appropriate.	Reporting the rationale for image selection helps readers understand the representativeness and educational value of the image while reducing potential selection bias.	
**Image Annotation**	8	Use arrows, labels, outlines, symbols, inset panels, scale bars, or other annotation tools when necessary to identify key findings and facilitate interpretation. All annotations should be explained in the figure legend.	Appropriate annotation guides readers to relevant findings, improves interpretability, and enhances the educational value of the image.	
**Image Description**	9	Provide a detailed and objective description of the visible findings, structures, abnormalities, or patterns demonstrated in the image.	Descriptions should focus on observable findings rather than diagnostic conclusions.	
**Diagnostic Interpretation**	10	Explain the clinical significance of the image findings and their contribution to diagnosis, differential diagnosis, management, or disease understanding.	Interpretation connects visual findings to their clinical implications and educational value.	
**Final Diagnosis**	11	State the confirmed diagnosis or the most probable diagnosis supported by available evidence.	Readers should be informed of the final diagnostic conclusion associated with the image.	
**Educational Message**	12	Present one or more key learning points emphasizing the educational relevance and practical implications of the image.	Clinical image publications should provide a concise and meaningful educational takeaway.	
**Ethics and Consent**	13	Confirm informed consent for publication when applicable and describe measures taken to protect patient privacy and confidentiality. Report ethical approval requirements according to local regulations and journal policies.	Ethical transparency is essential to ensure responsible publication and the protection of patient rights.	
**AI Use Disclosure**	14	Disclose whether artificial intelligence tools were used in image analysis, interpretation, annotation, enhancement, or manuscript preparation, and describe their role when applicable.	Transparency regarding AI use promotes reproducibility, accountability, and scientific integrity.	
**Figure Legend**	15	Provide a complete, accurate, and self-contained legend describing image content, relevant findings, annotations, abbreviations, and technical details required for interpretation.	A comprehensive legend allows readers to interpret the image independently of the main text.	

**Abbreviation:** IMAGE, Improving Clinical Image Reporting Standards. **Guidance for Authors:** Authors should indicate the page number(s) where each checklist item is addressed. If an item is not applicable, “N/A” should be reported with a brief justification when appropriate. **Guidance for Editors and Reviewers:** The IMAGE Checklist is intended to facilitate the evaluation of reporting completeness, transparency, educational value, scientific rigor, and ethical compliance in clinical image publications across healthcare disciplines.

## Data Availability

No new datasets were generated or analyzed during the current study. This work was based on a review of the published literature and publicly available journal instructions for authors. Therefore, data sharing is not applicable.

## Supplementary Materials

The following supporting information can be downloaded at https://www.mdpi.com/article/10.3390/mps9040121/s1, Table S1: Characteristics of journals accepting clinical images, image challenges, and short visual case reports.

## Abbreviations

The following abbreviations are used in this manuscript:

AI	Artificial Intelligence
CARE	CAse REport Guidelines
CLIP	Clinical and Laboratory Images in Publications
CONSORT	Consolidated Standards of Reporting Trials
EQUATOR	Enhancing the QUAlity and Transparency Of Health Research
IMAGE	Improving Clinical Image Reporting Standards
PRISMA	Preferred Reporting Items for Systematic Reviews and Meta-Analyses
